# RankAggreg, an R package for weighted rank aggregation

**DOI:** 10.1186/1471-2105-10-62

**Published:** 2009-02-19

**Authors:** Vasyl Pihur, Susmita Datta, Somnath Datta

**Affiliations:** 1 Department of Bioinformatics and Biostatistics, University of Louisville, Louisville, KY, USA

## Abstract

**Background:**

Researchers in the field of bioinformatics often face a challenge of combining several ordered lists in a proper and efficient manner. Rank aggregation techniques offer a general and flexible framework that allows one to objectively perform the necessary aggregation. With the rapid growth of high-throughput genomic and proteomic studies, the potential utility of rank aggregation in the context of meta-analysis becomes even more apparent. One of the major strengths of rank-based aggregation is the ability to combine lists coming from different sources and platforms, for example different microarray chips, which may or may not be directly comparable otherwise.

**Results:**

The *RankAggreg *package provides two methods for combining the ordered lists: the Cross-Entropy method and the Genetic Algorithm. Two examples of rank aggregation using the package are given in the manuscript: one in the context of clustering based on gene expression, and the other one in the context of meta-analysis of prostate cancer microarray experiments.

**Conclusion:**

The two examples described in the manuscript clearly show the utility of the *RankAggreg *package in the current bioinformatics context where ordered lists are routinely produced as a result of modern high-throughput technologies.

## Background

Rank aggregation has a long history with its roots tracing back to the voting theory of the 18^*th *^century. The Borda count is perhaps the most famous such method where elements in the overall list are ordered according to the average rank computed from the ranks in all individual lists. Other rank aggregation schemes have been proposed over the years and they differ greatly in both the underlying philosophy, as well as mathematical complexity.

Two radically different philosophies on rank aggregation exist. The first one is based on the majoritarian principles and attempts to accommodate the "majority" of individual preferences putting less or no weight on the relatively infrequent ones. The final aggregate ranking is usually based on the number of pairwise wins between items within individual lists. If item "A" is ranked higher than item "B" more often than not, then item "A" should also be ranked higher than item "B" in the overall list. Any method that satisfies this condition, known as the Condorcet criterion, is called the Condorcet method. The second philosophical approach to rank aggregation seeks the consensus among individual ordered lists and is usually based on some form of rank averaging. The Borda count is a good representative of this category. It is possible that the two approaches will produce different aggregated lists if applied to the same problem.

Conceptually, rank aggregation techniques range from quite simple (based on rank average or on a number of pairwise wins) to fairly complex which may employ advanced computational methodologies to find a solution. Simple solutions are not necessarily desirable as they usually rely on "ad hoc" principles and lack any formal justification. Mathematical rigor brings certain satisfaction and "security" at the expense of increased complexity and intensive computation.

Rank aggregation methods have a lot of potential in the field of bioinformatics. Ordered lists are routinely produced by today's high-throughput techniques which naturally lend themselves to a meta-analysis through rank aggregation. [1,2] proposed to use rank aggregation methods to integrate the results of several microarray studies (ordered lists of genes), [3,4] suggested aggregation of miRNA targets predicted by three popular software packages and [5] used rank aggregation to order clustering algorithms evaluated by several validation measures. The list can easily be extended to other potential applications, in particular, in proteomics for the purpose of integrating biomarkers from different studies or in the context of clustering analysis where the unknown number of clusters, instead of the "best" algorithm, needs to be determined. In this paper, we present an R *RankAggreg *package available through CRAN  which provides two different algorithms for rank aggregation: the Cross-Entropy Monte Carlo algorithm (CE) [6,7] and the Genetic algorithm (GA) [8]. Both methods are available through the main function *RankAggreg*. In addition, a brute force algorithm is also provided through the *BruteAggreg *function which simply tries all possible solutions and selects the one which is optimal. What is meant by "optimal" and how to find the "optimal" solution will be the discussion of the Methods section.

## Implementation

### Rank aggregation as an optimization problem

To cast the rank aggregation in the framework of an optimization problem, we first need to define the objective function. In this context, we would like to find a "super"-list which would be as "close" as possible to all individual ordered lists simultaneously. This is a natural requirement and the objective function, at least in its most abstract form, is very simple and intuitive

$$\Phi (\delta )=\sum_{i=1}^{m}w_{i}d(\delta ,L_{i}),$$

where *δ *is a proposed ordered list of length *k *= |*L*_*i*_|, *w*_*i *_is the importance weight associated with list *L*_*i*_, *d *is a distance function which will be discussed in details below, and *L*_*i *_is the *i*^*th *^ordered list [3,5].

The idea is to find δ* which would minimize the total distance between δ* and *L*_*i*_'s

$$\delta *=\arg\min\sum_{i=1}^{m}w_{i}d(\delta ,L_{i}).$$

Selecting the appropriate distance function *d *to measure the "distance" between ordered lists is very important. Though many choices for a distance function can be found in the literature, we concentrate on the two most popular ones: Spearman footrule distance and Kendall's tau distance. The two distances usually produce slightly different aggregated lists which is mainly due to the differences in the two philosophical paradigms discussed in the Background section.

### Spearman footrule distance

Before defining the two distance measures, let us introduce some necessary notations. Let *M*_*i*_(1),..., *M*_*i*_(*k*) be the scores associated with the ordered list *L*_*i*_, where *M*_*i*_(1) is the best (can be the largest or the smallest depending on the context) score, *M*_*i*_(2) is the second best, and so on. Let $r^{L_{i}}$ (*A*) be the rank of *A *in the list *L*_*i *_(1 means "best") if *A *is within the top *k*, and be equal to *k *+ 1, otherwise; *r*^*δ *^(*A*) is defined likewise. The Spearman's footrule distance between *L*_*i *_and any ordered list *δ *can be defined as

$$S(\delta ,L_{i})=\sum_{t\in L_{i}\cup \delta }|r^{\delta }(t)-r^{L_{i}}(t)|.$$

It is nothing more than the summation of the absolute differences between the ranks of all unique elements from both ordered lists combined. It is rather a very intuitive metric for comparing two ordered lists of arbitrary length. The smaller the value of the metric, the more similar the lists. For Spearman's footrule distance, the maximum value when comparing two top-*k *lists is *k*(*k *+ 1). It is attained when the two lists have no elements in common.

The appeal of the Spearman footrule distance comes from its simplicity and it is adequate in many situations when the only information available about the individual lists is the rank order of their elements. In a case when additional information which was used to rank the lists in the first place is available, it would be beneficial and prudent to incorporate this information into our aggregation scheme [5].

Thus, we define the Weighted Spearman's footrule distance between *L*_*i *_and any ordered list *δ *which makes use of the quantitative information available in many cases. It is given by this weighted sum representation

$$WS(\delta ,L_{i})=\sum_{t\in L_{i}\cup \delta }|M(r^{\delta }(t))-M(r^{L_{i}}(t))|\times |r^{\delta }(t)-r^{L_{i}}(t)|.$$

One can intuitively think of *WS*(*δ*, *L*_*i*_) in terms of sum of penalties for moving an arbitrary element of the list *L*_*i*_, *t*, from the position *r*^*δ *^(*t*) to another position $r^{L_{i}}$(*t*) within the list (second term of the products) adjusted by the difference in scores between the two positions (first term).

### Kendall's tau distance

The Kendall's tau distance takes a different approach at measuring the distance between two ordered lists. It utilizes pairs of elements from the union of two lists and is defined

$$K(\delta ,L_{i})=\sum_{t,u\in L_{i}\cup \delta }K_{tu}^{p},$$

where

$$K_{tu}^{p}=\{\begin{array}{ll} 0 & \text{if }r^{\delta }(t)<r^{\delta }(u),r^{L_{i}}(t)<r^{L_{i}}(u)\text{ or }r^{\delta }(t)>r^{\delta }(u),r^{L_{i}}(t)>r^{L_{i}}(u)\\ 1 & \text{if }r^{\delta }(t)>r^{\delta }(u),r^{L_{i}}(t)<r^{L_{i}}(u)\text{ or }r^{\delta }(t)<r^{\delta }(u),r^{L_{i}}(t)>r^{L_{i}}(u)\\ p & \text{if }r^{\delta }(t)=r^{\delta }(u)=k+1\text{ or }r^{L_{i}}(t)=r^{L_{i}}(u)=k+1. \end{array}$$

Here, *p *∈ [0, 1] is a parameter that needs to be specified for Kendall's tau. If *p *is set to 0, the maximum value that the distance can achieve is *k*^2 ^and this happens when the intersection of the two lists compared is an empty set. Intuitively, Kendall's tau can be thought about in the following way. If the two elements *t *and *u *have the same ordering in both lists, then no penalty is incurred (a good scenario). If the element *t *precedes *u *in the first list and *u *precedes *t *in the second list, then a penalty of 1 is imposed (a bad scenario). A case when both *t *and *u *do not appear in either one of the lists (their ranks are *k *+ 1) can be handled by selecting *p *on a spectrum ranging from very liberal (0) to very conservative (1). That is, if we have no knowledge of the relative position of *t *and *u *in one of the lists, we have several choices in the matter. We can either impose no penalty (0), full penalty (1), or a partial penalty (0 <*p *< 1). The following three choices are common: 0, 1, and 0.5. It is a matter of a philosophical taste as to which option one chooses. We use *p *= 0 in the internal Kendall function of the package.

Somewhat analogously to the Weighted Spearman distance, the Weighted Kendall's tau is defined by

$$WK(\delta ,L_{i})=\sum_{t,u\in L_{i}\cup \delta }|M(r^{L_{i}}(t))-M(r^{L_{i}}(u))|\times \;K_{tu}^{p},$$

in which the penalty imposed is adjusted by the absolute difference in the scores for elements *t *and *u*. Here, $K_{tu}^{p}$ is defined identically as above.

Normalization of scores from each list *L*_*i *_before computing *WS *and *WK *is necessary. The weights must be comparable otherwise disproportionately large or small weights can benefit a particular list and pull the "optimal" list δ* towards it. A number of normalization schemes that map the scores from the real line to the interval [0, 1] were considered. Unfortunately, most of them resulted in transformed scores occupying a very narrow portion of the interval. We settled for a simple normalization which spread the scores "evenly" between 0 and 1

$$M_{i}^{*}=\frac{M_{i}-\min(M_{i})}{\max(M_{i})-\min(M_{i})},i=1,...,n.$$

We would like to make one last comment on the reasons behind introducing weighted distance measures here. Quite obviously they are motivated by the desire for a more efficient use of the data, in this case, the numerical scores which underlie the rankings. But that is not their sole purpose. When using the original Spearman and Kendall distances we noticed that in many situations no clear winner exists as two or more ordered lists have the same objective function score due to the discrete nature of ranks. This brought computational instability into the iterative aggregation process. The algorithm would never converge but would simply oscillate between the two "best" lists, understandably not knowing which one to pick. When continuous weights are used to adjust the discrete ranks, the possibility of such ties is almost eliminated and the algorithm is much more computationally stable. In addition, we obtain a clear winner in an objective and rigorous way.

### Cross-Entropy Monte Carlo algorithm

The details of the CE algorithm are given in [5]. [3] explore the CE algorithm for rank aggregation in the context of microRNA analysis and a very useful tutorial on the CE algorithm with several examples is presented in [7]. Here, we only briefly describe the main steps. The CE is a stochastic search algorithm in the space of matrices with 0–1 valued entries with columns summing to one and rows summing to at most one since any ordered list can be uniquely mapped to such a matrix.

1. **Initialization: **Start with the uniform multinomial cell probabilities.

2. **Sampling: **At each stage, generate a random sample of such matrices via restricted (truncated) multinomial sampling with the current cell probabilities.

3. **Updating: **Based on the current sample and the value of the objective function at the corresponding ordered list update the multinomial cell probabilities such that the objective functions at the next batch of sample values tend to be smaller.

4. **Convergence: **Stop the search when the smallest values of the objective function do not change in a given number of iterations.

The CE algorithm requires users to set a number of parameters. Convergence to a global optimal solution in many ways depends on the parameters chosen. It is recommended that the number of samples *N *for each stage is to be set to at least 10*k*^2 ^(in case, *n *>> *k*, 10*kn*, where *n *is the total number of unique elements being ordered *k *at a time) and the rarity parameter *ρ *used in updating the cell probabilities is to be set to 0.01 if *N *is relatively large or 0.1 if *N *is small (less than 100).

### Genetic algorithm

Genetic algorithms are another set of tools suitable for solving complex combinatorial problems [8]. Their main advantage is their inherent simplicity in both conceptual understanding and software implementation. In our experience, the GA performs reasonably well for the aggregation problem but one has to be careful with the selection of important tuning parameters which control the rate of the learning process.

As implemented in this package, the GA has the following steps:

1. **Initialization: **Randomly select *popSize *ordered lists of size *k *which form the initial population of possible solutions to our optimization problem. The population size *popSize *is important and, obviously, the larger the population size, the better chance of it containing, at some point, the optimal solution. It should ideally be a function of *k *and the number of unique elements in the original ordered lists *L*_*i*_, but computational feasibility has to be considered here.

2. **Selection: **Depending on which distance is used, compute the objective function for each member of the population. Then randomly select current members for the next generation using weighted random sampling where the weights are determined by the member's fitness (the objective function score).

3. **Cross-over: **The selected members are then crossed-over with the probability of *CP *(the cross-over probability), i.e. two random ordered lists can swap their tails which start at a random position with the *CP *probability. Only 1-point cross-overs are allowed.

4. **Mutation: **Crossing-over will allow only for the mixing of ordered lists but a rather drastic event is required to bring radically new solutions to the population pool. These are introduced by mutations which happen with the probability of MP (mutation probability). Thus, any list in the pool can randomly change one or more of its elements.

5. **Convergence: **The algorithm is stopped if the "optimal" list remains optimal for *convIn *consecutive generations (default is 15). To ensure that the algorithm stops running eventually, the maximum number of generations can be set in advance which will terminate the execution regardless of the first condition being true. If neither the maximum number of iterations has been reached nor the "optimal" list stayed untouched during the last *convIn *generations, continue to step Selection.

As was mentioned previously, the choice of the parameters *popSize*, *CP*, and *MP *is crucial for the success of the GA. If one is too conservative and selects small *CP *and *MP *probabilities, the GA will have a hard time exploring the space of possible solutions in a reasonable time, particularly, when the space is extremely large. On the other hand, choosing large values for *CP *and *MP *will results in a "haste" decision, perhaps getting trapped in a local minimum without a chance to explore the whole search space.

## Results and Discussion

We illustrate our R package with two different rank aggregation problems, one in the context of unsupervised learning where there is an intrinsic difficulty of choosing the best clustering algorithm for a particular problem, and another one in the context of meta-analysis of several microarray cancer studies where the goal is to determine the combined set of genes indicative of the cancer status.

To start using the *RankAggreg *package, it must be loaded into R [9] with the regular *library( ) *function, *library(RankAggreg)*. Package documentation, examples, and additional information are available through *help(package = "RankAggreg") *and *vignette("RankAggreg") *functions.

### Aggregation of clustering validation measures

Rank aggregation in the clustering context was introduced by [5]. Numerous clustering algorithms are available in R and other statistical and data mining software packages, each one having its relative strengths and weaknesses in terms of how successfully they can handle certain types of data. Thus, it is often difficult to select the "best" algorithm for a particular clustering task. Validation (performance) measures come to rescue to some extent and offer an objective way of ranking clustering algorithms according to their assessment of what a "good" clustering result is. If *k *clustering algorithms are validated with *m *validation measures, *m *ordered lists of size *k *are produced as a result. Even though desirable, the order of clustering algorithms within each list is rarely the same. Rank aggregation is helpful in reconciling the ranks and producing the "super"-list which determines the overall winner and also ranks all clustering algorithms based on their performance as determined by all *m *validation measures simultaneously. Clustering validation is implemented in the *clValid *package [10]. After loading the package, we bring in a mouse microarray dataset and select the first 100 genes from it. Assuming that those 100 genes form 5 natural clusters (this is an ad-hoc assumption but it is not essential for the rank aggregation demonstration), we evaluate 10 clustering algorithms with 6 validation measures. Available clustering algorithms are: SOM (SM), SOTA (ST), FANNY (FN), K-Means(KM), PAM(PM), Hierarchical(HR), Agnes(AG), CLARA(CL), Diana(DI), and Model-based(MO). Further details can be obtained from the *clValid *package documentation.

For each validation measure, 10 clustering algorithms can now be ranked based on the performance scores which are sorted either in ascending or descending order depending on whether larger or smaller scores correspond to better performance under the measure. Here, the Dunn index and the Silhouette Width measures give higher scores with better performance and for the other measures the smaller scores are desirable.

The 7 ordered lists of 10 algorithms are shown in Table 1. Their corresponding weights (validation measure scores) which were used to rank the 10 algorithms within each ordered list (in rows) are shown in Table 2. We can see that both SOM and Hierarchical clustering are performing quite well and each is ranked first by three different validation measures. If we had to pick the overall winner, it would probably be SOM as it performs better overall. For this particular aggregation problem it is feasible to determine the best performer without resorting to advanced computational techniques, but it is rather difficult to obtain the overall ordered list in this case. Since the number of possible solutions is not that large (*k*! = 10! = 3, 628, 800), it is feasible to use the brute force approach to find the optimal solution. This can be done using the *BruteAggreg( ) *function provided in the package. Please note that even for this relatively small problem it takes hours to perform the necessary computations. The approach is limited to toy examples only and should not be attempted if *k *is larger than 10.

**Table 1:** Clustering algorithms ranks

	1	2	3	4	5	6	7	8	9	10
APN	SM	FN	ST	KM	PM	HR	AG	CL	DI	MO
AD	SM	FN	KM	PM	CL	ST	DI	HR	AG	MO
ADM	FN	SM	ST	KM	CL	PM	DI	HR	AG	MO
FOM	SM	CL	KM	PM	FN	ST	DI	HR	AG	MO
Connectivity	HR	AG	DI	KM	MO	SM	FN	CL	PM	ST
Dunn	HR	AG	KM	PM	DI	SM	CL	MO	FN	ST
Silhouette	HR	AG	KM	SM	CL	PM	ST	DI	FN	MO

10 clustering algorithms ranked by 7 validation measures (in rows). The rank of 1 means that the algorithm received the best scored from a particular validation measure. For example, SOM is deemed to be the best algorithm by APN, AD, and FOM measures, while MO is ranked last by 5 out of 7 measures.

**Table 2:** Validation scores

	1	2	3	4	5	6	7	8	9	10
APN	0.11	0.12	0.15	0.15	0.17	0.17	0.17	0.18	0.18	0.26
AD	1.63	1.67	1.70	1.71	1.71	1.74	1.83	1.85	1.85	2.50
ADM	0.28	0.31	0.37	0.47	0.48	0.48	0.57	0.63	0.63	0.85
FOM	0.57	0.58	0.58	0.59	0.59	0.59	0.60	0.68	0.68	0.80
Connectivity	23.91	23.91	35.44	36.09	37.49	38.40	38.82	39.58	39.84	49.93
Dunn	0.17	0.17	0.12	0.11	0.11	0.11	0.08	0.08	0.08	0.06
Silhouette	0.39	0.39	0.38	0.36	0.35	0.35	0.33	0.31	0.30	0.16

Clustering validation scores for each validation measure as produced by the *clValid *package. Please note that the rows are ordered in either ascending or descending order depending whether larger or smaller scores are desirable for a particular validation measure.

The R code to perform the brute force rank aggregation is

> BruteAggreg(ranks, 10, weights, "Spearman")

Here, the first argument is the matrix of ordered lists (in rows), the second argument is the size of the resulting top list which is 10 in this case, the third argument specifies the matrix of weights which is normalized by the procedure (note that there is no need to worry about mixing both ascending and descending orders; the only requirement is that the rows must be sorted), and the last argument indicates that we want to use the Spearman footrule distance as the measure of similarity between two ordered lists. The best overall list as determined by trying all possible solutions with the weighted Spearman footrule distance is SM HR KM FN AG PM CL DI ST MO with the minimum objective function score of 5.552256. As expected, SOM and Hierarchical clustering are the top two performers, followed by the K-Means algorithm. We will now see whether the CE algorithm can quickly discover the solution without resorting to an exhaustive search.

**Figure 1:**
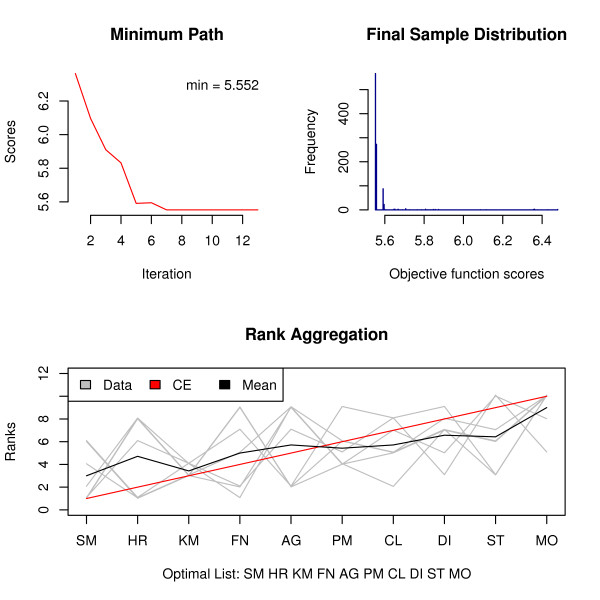
**Rank aggregation in the clustering context using the CE algorithm**. Visual Representation of the aggregation results through the *plot( ) *function for the Clustering example using the CE algorithm and the Spearman footrule distance. The first plot in the top row shows the path of minimum values of the objective function over time. The global minimum is shown in the top right corner. The histogram of the objective function scores at the last iteration is displayed in the second plot. Looking at these two plots, one can get a general idea about the rate of convergence and the distribution of candidate lists at the last iteration. The third plot at the bottom shows the individual lists and the obtained solution along with optional average ranking.

The *RankAggreg( ) *function performs rank aggregation using either the CE algorithm or the GA algorithm. If the *verbose *argument of the *RankAggreg( ) *function is set to TRUE (it is by default), R console window outputs information at each iteration to keep the user updated. In addition, a plot similar to Figure 1 is shown and updated at each iteration to monitor convergence.

Running the following code in R

> RankAggreg(ranks, 10, weights, seed = 123)

performs the rank aggregation using the CE algorithm. We get exactly the same solution in only 13 iterations and in about 40 seconds by examining mere 13000 potential candidates. The CE algorithm was run 20 times using the default values with 20 different seeds. Only 1 out of 20 times it failed to discover the optimal solution, switching the K-Means and SOM algorithms.

To get a visual representation of the results, a convenient *plot( ) *function is provided. It takes the object returned by the *RankAggreg( ) *function as its first argument and outputs three side-by-side plots with useful information on the convergence properties and the final ranking. From these plots we see that already after 7 iterations the CE algorithm found the optimal solution. The distribution of the final Monte Carlo sample is shown in the second plot. Most of the mass is put on the optimal value (most of the candidate lists are the same "optimal" list). The last plot visualizes ordered lists to be combined and the resulting solution.

Figure 1: Visual Representation of the aggregation results through the *plot( ) *function. The first plot in the top row shows the path of minimum values of the objective function over time. The global minimum is shown in the top right corner. The histogram of the objective function scores at the last iteration is displayed in the second plot. Looking at these two plots, one can get a general idea about the rate of convergence and the distribution of candidate lists at the last iteration. The third plot at the bottom shows the individual lists and the obtained solution along with optional average ranking.

Weighted Kendall's tau distance can also be used, though it is much more expensive to compute.

> RankAggreg(ranks, 10, weights, "CE", "Kendall", seed = 123)

The overall list is given by KM SM PM FN HR AG CL DI ST MO with the value of 1.241372. Thus, the SOM is put in the second position with K-Means occupying the first place. Maybe somewhat surprisingly, the Hierarchical clustering (HR) algorithm is ranked fifth despite the fact that it was ranked number 1 in 3 out of 7 lists. The rational explanation behind this decision is given by its poor performance according to the other four measures which rank it towards the end.

**Figure 2:**
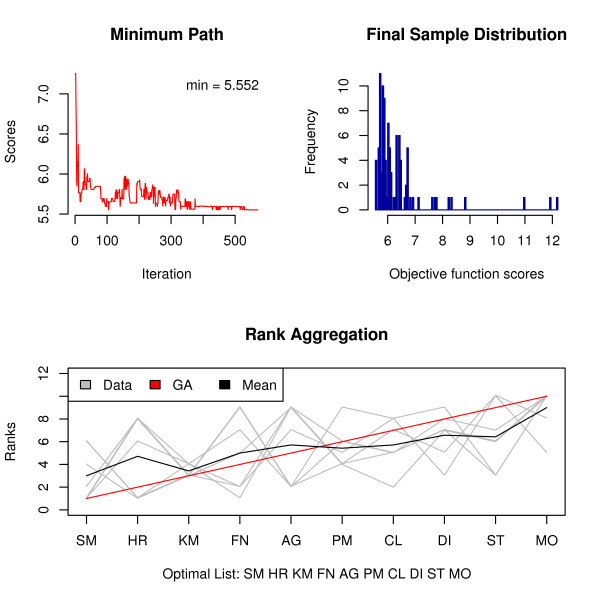
**Rank aggregation in the clustering context using the GA algorithm**. Visual representation of rank aggregation for the Clustering example using the GA algorithm with the Weighted Spearman distance.

The Genetic Algorithm can also be used with either one of the two distance measures. Both results agree with the ones obtained using the CE algorithm. Besides the jaggedness of the minimum path in the first plot of Figure 2, it is easy to notice that the GA algorithm takes significantly larger amount of cycles to converge but they take less time to complete. Even given that, the population distribution of the last generation is much more heterogeneous than that of the CE.

Figure 2: Visual representation of rank aggregation using the GA algorithm with the Weighted Spearman distance.

### Meta-analysis of microarray experiments

Microarray cancer studies often attempt to identify genes related to a specific cancer. Their most common output is a list of genes ordered by corresponding p-values. Different studies, even the ones analyzing the same cancer type (for example, lung cancer), almost never produce identical gene lists. Meta-analysis of multiple microarray studies is difficult, especially if different experimental platforms have been used. Rank aggregation, however, avoids the issue of multiple experimental conditions by dealing with the final product: the ordered list of genes.

Recently, we carried out a meta-analysis of 20 microarray studies on multiple cancers using the proposed rank aggregation algorithms [2]. Our goal was to identify genes which would be important in development of multiple cancers. Further details can be found in the original article. Here, we present a smaller example described by [1] who used three different Monte Carlo algorithms for rank aggregation of 5 prostate cancer microarray datasets. Two experiments were conducted using the Affymetrix chip technology and the other three studies used custom cDNA chips. Each individual study tried to identify genes which are either up or down-regulated in prostate cancer patients, resulting in ordered lists of upregualated genes shown in Table 3 (the lists appear in Table 4 in [1]).

**Table 3:** Top-25 prostate cancer gene lists

	Luo	Welsh	Dhana	True	Singh
1	HPN	HPN	OGT	AMACR	HPN
2	AMACR	AMACR	AMACR	HPN	SLC25A6
3	CYP1B1	0ACT2	FASN	NME2	EEF2
4	ATF5	GDF15	HPN	CBX3	SAT
5	BRCA1	FASN	UAP1	GDF15	NME2
6	LGALS3	ANK3	GUCY1A3	MTHFD2	LDHA
7	MYC	KRT18	0ACT2	MRPL3	CANX
8	PCDHGC3	UAP1	SLC19A1	SLC25A6	NACA
9	WT1	GRP58	KRT18	NME1	FASN
10	TFF3	PPIB	EEF2	COX6C	SND1
11	MARCKS	KRT7	STRA13	JTV1	KRT18
12	OS-9	NME1	ALCAM	CCNG2	RPL15
13	CCND2	STRA13	GDF15	AP3S1	TNFSF10
14	NME1	DAPK1	NME1	EEF2	SERP1
15	DYRK1A	TMEM4	CALR	RAN	GRP58
16	TRAP1	CANX	SND1	PRKACA	ALCAM
17	FM05	TRA1	STAT6	RAD23B	GDF15
18	ZHX2	PRSS8	TCEB3	PSAP	TMEM4
19	RPL36AL	ENTPD6	EIF4A1	CCT2	CCT2
20	ITPR3	PPP1CA	LMAN1	G3BP	SLC39A6
21	GCSH	ACADSB	MAOA	EPRS	RPL5
22	DDB2	PTPLB	ATP6V0B	CKAP1	RPS13
23	TFCP2	TMEM23	PPIB	LIG3	MTHFD2
24	TRAM1	MRPL3	FM05	SNX4	G3BP2
25	YTHDF3	SLC19A1	SLC7A5	NSMAF	UAP1

Top-25 upregulated genes from 5 different prostate microarray experiments (as reported in [1]). HPN is the sole gene that appears in all five lists.

As shown in Table 3, there are 89 unique genes in all 5 gene lists. The only gene that appears in all of them is HPN, while genes AMACR, GDF15, and NME1 appear in 4 lists. 66 genes appear in just one list. The goal of rank aggregation is to combine these lists into the overall top-25 gene list which hopefully would be more accurate than any individual list by itself.

Since no p-values were reported, we use the regular Spearman distance for both the CE and the GA algorithms.

> data(geneLists)

> RankAggreg(geneLists, 25, seed = 100, rho = 0.01)

Using the CE algorithm with the Spearman distance, the following ordered list was produced: HPN AMACR GDF15 FASN NME2 UAP1 SLC25A6 0ACT2 KRT18 NME1 EEF2 STRA13 GRP58 CANX SND1 ALCAM MRPL3 TMEM4 CCT2 MTHFD2 SLC19A1 PPIB FM05 ENTPD6 KRT7. The algorithm converged in 38 iterations with the minimum of 319.6. In the overall list, HPN, as expected, is in the first place, followed closely by the two other genes that appear in four lists.

In a case when there would be an indication that some microarray studies are more reliable than others, we could set the *importance *parameter available in the *RankAggreg *function to reflect these beliefs. By default, it assigns equal weights to all ordered lists, but one, for example, could set *importance *= *c*(1, 2, 1, 1, 2) placing stronger emphasis on the Affymetrix arrays which are considered to have higher sensitivity rates.

> RankAggreg(geneLists, 25, seed = 100, importance =

+ c(1,2,1,1,2), rho = 0.01)

This produces the following combined list which is slightly different from the one obtained treating all five studies equally:

HPN AMACR 0ACT2 GDF15 FASN NME2 KRT18 SLC25A6 EEF2 UAP1 CANX NME1 GRP58 SND1 STRA13 TMEM4 ALCAM PPIB NACA CCT2 RPL5 SLC39A6 MTHFD2 MRPL3 SLC19A1.

The objective function score here is 295.43, being a little smaller than 319.6. Clearly, OACT2 is ranked higher now (3rd) due to being at the top (also 3rd) in the Welsh study which received more weight. Similarly, the KRT18 gene moved up a couple spots due to being present in both Welsh and Singh top lists which are both Affymetrix.

The GA algorithm can also be applied. We increased the maximum number of iterations to allow for a longer evolution process. Increasing the *convIn *(converge in) argument to 50 will assure that we do not stop the algorithm too soon. The algorithm did not converge (due to setting a rather stringent criteria) and was stopped after 3000 generations. The final list had an objective function score of 320.8, which was slightly worse than what we obtained using the CE algorithm. Here is the list found by the GA algorithm:

HPN AMACR SLC25A6 FASN NME2 GDF15 0ACT2 UAP1 KRT18 EEF2 STRA13 NME1 MTHFD2 SND1 CANX GRP58 ALCAM TMEM4 PPIB CCT2 SLC19A1 CBX3 SAT FM05 SNX4.

Based on the value of the objective function, we prefer the list identified by the CE algorithm in this case. From that list, 9 genes were previously linked to prostate cancer development in the literature: HPN, AMACR, GDF15, FASN, SLC25A6, KRT18, ALCAM, CCT2, and MTHFD2. Note that 6 of them are among the top-10 genes in the obtained list. [11] find strong evidence for HPN's association with prostate cancer susceptibility and tumor aggressiveness, AMACR was shown to be overexpressed in prostate cancer by [12], and [13] propose to use FASN, which is also overexpressed, as a therapeutic target for prostate cancer. If we, for example, ignored the overall list for a moment and concentrated on the individual lists, we would miss a clearly important gene FASN 2 out of 5 times as it does not appear in Luo and True top-25 lists. The overall list, which borrows the information across the studies, places FASN in the fourth place making it impossible to overlook.

Lin and Ding [4] also used the CE algorithm to aggregate the same five gene lists with the results presented in Table 2 in their original manuscript. The obtained list for the unweighted Spearman column is very similar to the one obtained using the *RankAggreg( ) *function. On their supplementary website, the authors made available an R function that performs rank aggregation using the CE algorithm; the underlying code is written in C. At this time, it is somewhat faster than the *RankAggreg( ) *function but it lacks the user-friendliness and exibility that our package offers. In addition, it provides no alternatives to the CE algorithm and does not make use of the weighted distance functions that we proposed.

To give a reader some perspective on whether the computational overhead is well justified in this case, we aggregated the five lists using the Borda count method described in the Background section. According to this simple procedure, the overall list is

HPN AMACR GDF15 FASN NME1 EEF2 KRT18 NME2 0ACT2 SLC25A6 UAP1 CANX GRP58 STRA13 SND1 OGT ALCAM CYP1B1 MTHFD2 ATF5 CBX3 SAT BRCA1 MRPL3 ANK3.

The first four genes are the same when compared to the result obtained using the CE algorithm with equal weights. The order of other genes, however, is different. The lists share 18 common genes among the two of them. Using the Spearman distance, we calculated the objective function score for the list obtained from the Borda count and it turned out to be 333.6, which is a little worse than 319.6 that we get using the CE algorithm. Thus, the extra cost in computing may be well justified as we obtained a better list with a smaller score.

**Figure 3:**
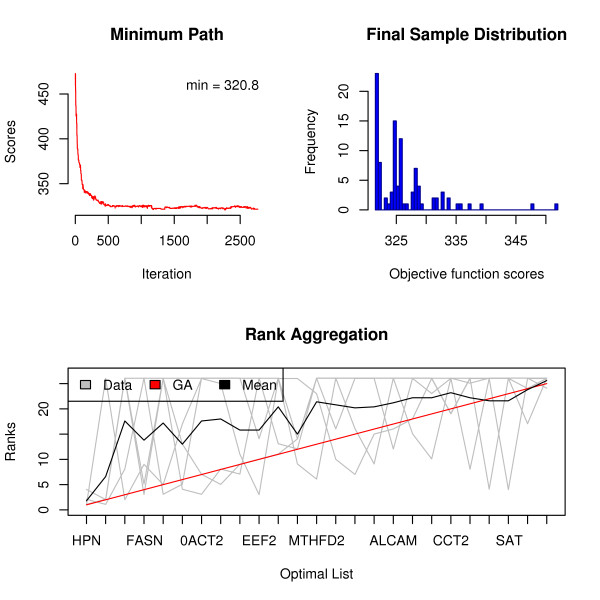
**Rank aggregation of gene lists using the GA algorithm**. Plots created by the *plot( ) *function for the GA rank aggregation of the gene lists. We can see that algorithm stabilized after roughly 500 iterations. The distribution of the population in the final generation is concentrated just to the right of the optimal solution. The bottom plot clearly shows why the solution makes sense. Genes ranked high in the final list usually come from several individual lists as indicated by the presence of multiple lines intersecting. The genes at the end of the final list are the ones included in a single list but somewhere close to the top. The rank of 26 is artificial in our procedure and it simply indicates that that particular gene is not present in the individual list.

Figure 3: Plots created by the *plot( ) *function for the GA rank aggregation of the gene lists. We can see that algorithm stabilized after roughly 500 iterations. The distribution of the population in the final generation is concentrated just to the right of the optimal solution. The bottom plot clearly shows why the solution makes sense. Genes ranked high in the final list usually come from several individual lists as indicated by the presence of multiple intersecting lines. The genes at the end of the final list are the ones included in a single list but somewhere close to the top. The rank of 26 is artificial in our procedure and it simply indicates that that particular gene is not present in the individual list.

## Conclusion

The *RankAggreg *package provides an easy and convenient interface to handle complex rank aggregation problems. It performs rank aggregation using two different algorithms with a choice of two different distances. The brute force approach is also available for small-scale problems. A simple plot function helps to visualize the rank aggregation problem and the obtained solution.

The effectiveness of the CE and the GA algorithms in discovering optimal lists is certainly limited by the size of the aggregation problem. As both algorithms need to effectively search the solution space, which even in moderate aggregation problems (for example, discovering a top-25 list) is extremely large, there exists a practical limitation as to what problems can be handled. The examples presented in this article are rather of a moderate size but larger problems can definitely be tackled. Top-100 lists with a significant amount of overlap in terms of their content can certainly be aggregated using either one of the proposed algorithms. How well they will perform in aggregation of much larger problems remains to be investigated. In the bioinformatics context, however, researchers are often interested in a relatively small number (20–50) of significant discoveries and their aggregation is within the limits of the proposed methodology.

We would like to stress that using either the CE or the GA algorithms for large problems does not "guarantee" an optimal solution. Performance of both of these algorithms is quite sensitive to the tuning parameters, in particular the sample size *N *for the CE algorithm and the cross-over (CP) and mutation (MP) probabilities for the GA algorithm. The user is encouraged to run the *RankAggreg( ) *function several times. If different optimal lists are produced, increasing sample size is probably necessary. Tuning additional parameters as discussed above may also prevent local minima traps. That said, however, we are quite impressed by the ability of both algorithms, the CE in particular, in discovering the optimal ordering of the elements in the combined list.

## Availability and requirements

Project name: RankAggreg

Project home page: 

Operating system(s): Windows, Unix

Programming language: R

Other requirements: R-2.4.0 or newer

License: LGPL

The *RankAggreg *package can be installed from the CRAN using the *install.packages("RankAggreg") *command. The local zip file can be installed using R GUI by selecting Packages and then Install package(s) from local zip files.

## Authors' contributions

VP produced the *RankAggreg *package and contributed towards planning and writing of the manuscript, particularly producing the Results section. SD and SD provided guidance and planning for the project, and contributed towards writing the manuscript.
